# The interplay of prenatal stress and prenatal depression in Chinese couples: based on the actor-partner interdependence model

**DOI:** 10.3389/fpsyt.2025.1607470

**Published:** 2025-07-31

**Authors:** Xiaoqing Liu, Yuting Dong, Jungu Zhou, Shiying Lin, Minghuan Liu, Hang Zeng, Xiaowan Zhou, Yang Song, Xiaoxu Xie, Qiuping Liao, Shaowei Lin, Siying Wu

**Affiliations:** ^1^ Department of Epidemiology and Health Statistics, School of Public Health, Fujian Medical University, Fuzhou, Fujian, China; ^2^ Fujian Maternity and Child Health Hospital, Fuzhou, Fujian, China

**Keywords:** actor-partner interdependence model (APIM), patterns, prenatal stress, prenatal depression, couples’ mental health

## Abstract

**Objective:**

Pregnant women exhibit heightened stress susceptibility and elevated depression risk during gestation, factors associated with adverse outcomes including postpartum depression. Current research predominantly examines maternal experiences while neglecting spousal influences.

**Methods:**

The study surveyed 282 Chinese married couples using validated scales to assess prenatal stress and depression. And analysed dyadic data from expectant parents using the Actor-Partner Interdependence Model (APIM) to determine the pattern of action of prenatal stress on prenatal depression between couples by calculating the magnitude of the ratio *k* between the partner effect and the actor effect.

**Results:**

The analysis revealed *k_1_* = 0.064, 95% *CI:* (-0.113, 0.260) and *k_2_* = 0.064, 95% *CI*: (-0.118, 0.249). The confidence intervals for both *k_1_* and *k_2_* included zero, indicating an actor-only pattern in the APIM. Specifically, prenatal stress positively predicted one’s own prenatal depression but did not significantly influence the partner’s depression.

**Conclusion:**

It is crucial to encourage couples to actively manage their stress levels during the prenatal period. This can help to reduce the negative psychological effects of prenatal stress, which may lead to improved pregnancy outcomes and postnatal health.

## 1 Introduction

Pregnant women frequently experience psychiatric symptoms such as anxiety and depression during gestation, attributable to physiological adaptations, psychological stressors, and evolving familial/social roles (1). While the postpartum period has traditionally dominated research on maternal mental health (2), emerging evidence identifies that prenatal depression is a potent predictor of postpartum depression and is a more prevalent condition (3), with estimated prevalence rates of 7-20% in high-income countries and ≥20% in low- and middle-income nations (4). This condition is linked to adverse pregnancy outcomes, with long-term consequences extending into childhood and adulthood (5, 6). Notably, expectant fathers also face underrecognized emotional challenges during his wife’s pregnancy, including childbirth-related anxieties, and ambivalence toward pregnancy, which may precipitate depressive symptoms (7). A meta-analysis revealed a 9.76% prevalence of paternal prenatal depression throughout pregnancy, peaking at 13.59% during early gestation (8). Crucially, paternal depression negatively impacts maternal mental health and indirectly affects fetal/neonatal outcomes (9). The stress-depression nexus is well-established in epidemiological research (10), with substantial evidence linking prenatal stress exposure to elevated prenatal depression risk (11). However, existing research predominantly examines pregnant women individually, neglecting the dyadic relationships within couples.

**Figure 1:**
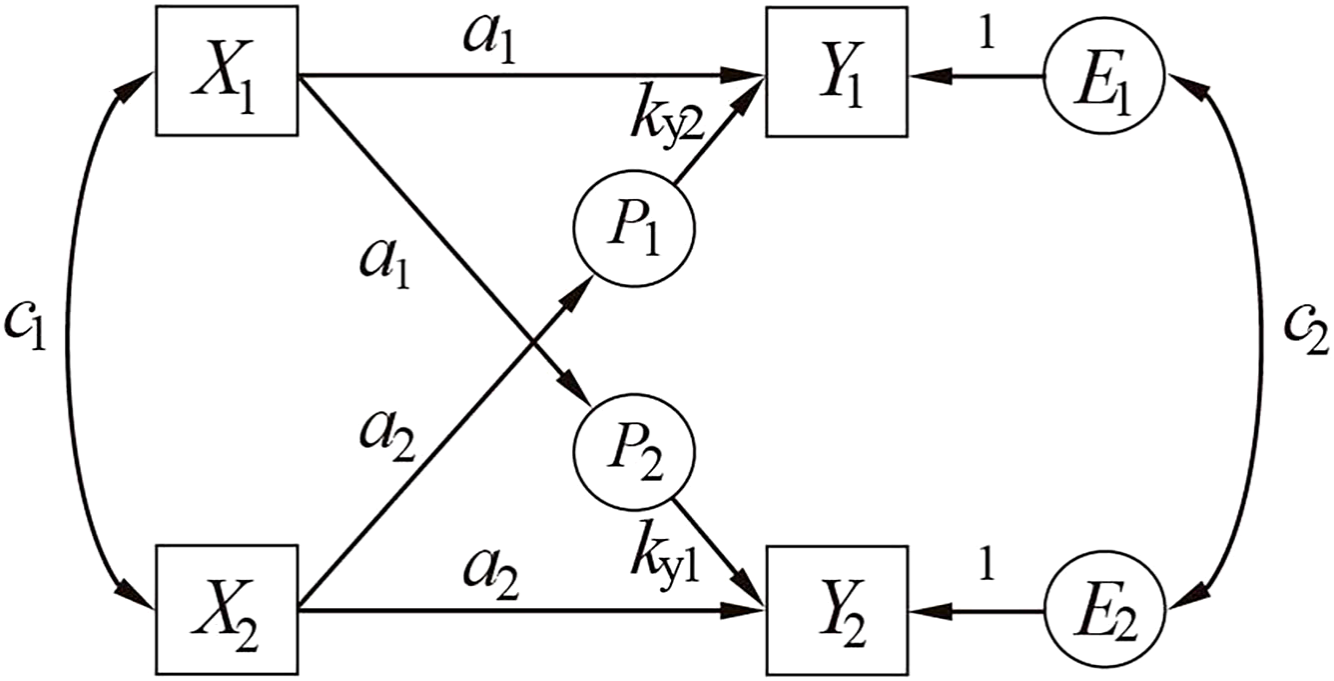
The actor-partner interdependence model. *X*_1_ /*X*_2_, *Y*_1_ /*Y*_2_ = measured variables; *E*_1_ /*E*_2_ = error terms; *a*= actor effect; *p* = partner effect; *c*_1_ /*c*_2_ = correlated residuals.

The Actor-Partner Interdependence Model (APIM), introduced by Kenny and Cook (12), serves as the gold standard for dyadic data analysis (**Figure 1**). This framework accounts for dyadic data non-independence and has become an established method in marital, familial, and behavioral research (13). The APIM partitions relational effects into actor effects (*a*), where predictors influence one’s own outcomes, and partner effects (*p*), where predictors affect a partner’s outcomes (14). Building on this framework, Kenny et al. (15) delineated four interaction patterns: (1) actor-only (*a*≠0, *p*=0: self-predictors exclusively impact personal outcomes), (2) partner-only (*a*=0, *p*≠0: predictors solely influence partner outcomes), (3) couple-oriented (*a*=*p*: bidirectional equivalent effects), and (4) contrast (*a*=−*p*: opposing self/partner predictive directions).

Family system theory proposed that family relationships and dynamics are interactive, and each of the relationships is depending on each other (16). This fits perfectly with APIM’s assumption. Besides, Stress can be considered a dyadic phenomenon- one’s external stress can affect not only one’s own perceived relationship stress (actor effects) but also the other partner’s perceived relationship stress (partner effect) (17). This interpersonal transmission mechanism renders stress uniquely suited for analysis via APIM. Thus, this study examines whether partners’ prenatal stress exhibits cross-spousal influences on depression development using APIM, aiming to identify predominant dyadic effect patterns. The findings will provide empirical evidence to guide targeted interventions for enhancing dual-partner prenatal mental health.

## 2 Materials and methods

### 2.1 Study design

This cross-sectional study applied the Actor-Partner Interdependence Model (APIM) to determine the pattern of action of prenatal stress on prenatal depression between couples by calculating the ratio *k* of the partner effect to the actor effect.

### 2.2 Participants

A convenience sample of 340 couples meeting inclusion criteria was recruited from Provincial Maternal and Child Health Hospital, China (December 2023-April 2024). Pregnant women’s electronic medical record data were sourced from this hospital. Eligibility required: gestational age ≥28 weeks; absence of mental health diagnoses; survey comprehension capacity; and voluntary participation. Exclusion criteria included: incomplete data submission; withdrawal of consent; severe physical/mental conditions preventing independent participation; and communication barriers. From the 335 pregnant women and 296 spouses who completed surveys, 282 valid couple-dyad questionnaires were retained after excluding invalid responses, yielding an 82.74% validity rate. The study obtained written informed consent from all participants.

Following eligibility screening and informed consent, trained researchers detailed the study objectives and procedures. Participants completed self-administered questionnaires on-site under direct supervision. Spouses completed questionnaires independently in separate rooms without communication, with immediate collection upon completion to ensure data integrity. We emphasized anonymity and confidentiality throughout, enabling participants to express feelings freely and enhancing data reliability.

### 2.3 Measures

#### 2.3.1 General information questionnaire

A structured socio-demographic questionnaire was developed through literature synthesis aligned with study objectives, capturing key variables including gender, age, employment status, and gestational age.

#### 2.3.2 Prenatal stress

The 14-item Perceived Stress Scale (PSS-14) (18), cross-culturally adapted for Chinese populations (19) was utilized to measure psychological stress. Responses were recorded on a 5-point Likert scale (0=never to 4=very often), with positive subscale items reverse-scored. Total scores range from 0 to 56, with higher scores indicating greater perceived stress (20). In this study, the Chinese PSS-14 demonstrated strong reliability in this cohort (Cronbach’s α=0.81 pregnant women, 0.85 partners).

#### 2.3.3 Prenatal depression

Depressive symptoms were assessed using the Chinese version of the Edinburgh Postnatal Depression Scale (EPDS) (21), a validated 10-item self-report measure for perinatal populations. Items evaluate symptom severity on a 4-point scale (0-3), yielding total scores 0–30 with higher scores indicating more severe antenatal depression (clinical cutoff ≥10) (22) The instrument showed excellent internal consistency in this study (Cronbach’s α=0.82 pregnant women, 0.85 partners).

### 2.4 Statistical methods

#### 2.4.1 Distinguishable and indistinguishable dyadic data

Dyads are distinguishable when partners have unique roles (e.g., pregnant woman vs. spouse) and indistinguishable without systematic role differentiation. We simultaneously fit two nested models: an unconstrained (saturated) model allowing actor and partner effects to vary freely across partners, and a constrained model equating actor and partner effects. A likelihood ratio test for model comparison produced chi-square and p-values. When *P* ≥ 0.2, indistinguishability is confirmed and the constrained model retained; when *P* < 0.2, distinguishability is confirmed and the unconstrained model adopted (15).

#### 2.4.2 Definition, estimation, and confidence intervals of the ratio *k*

**Figure 2:**
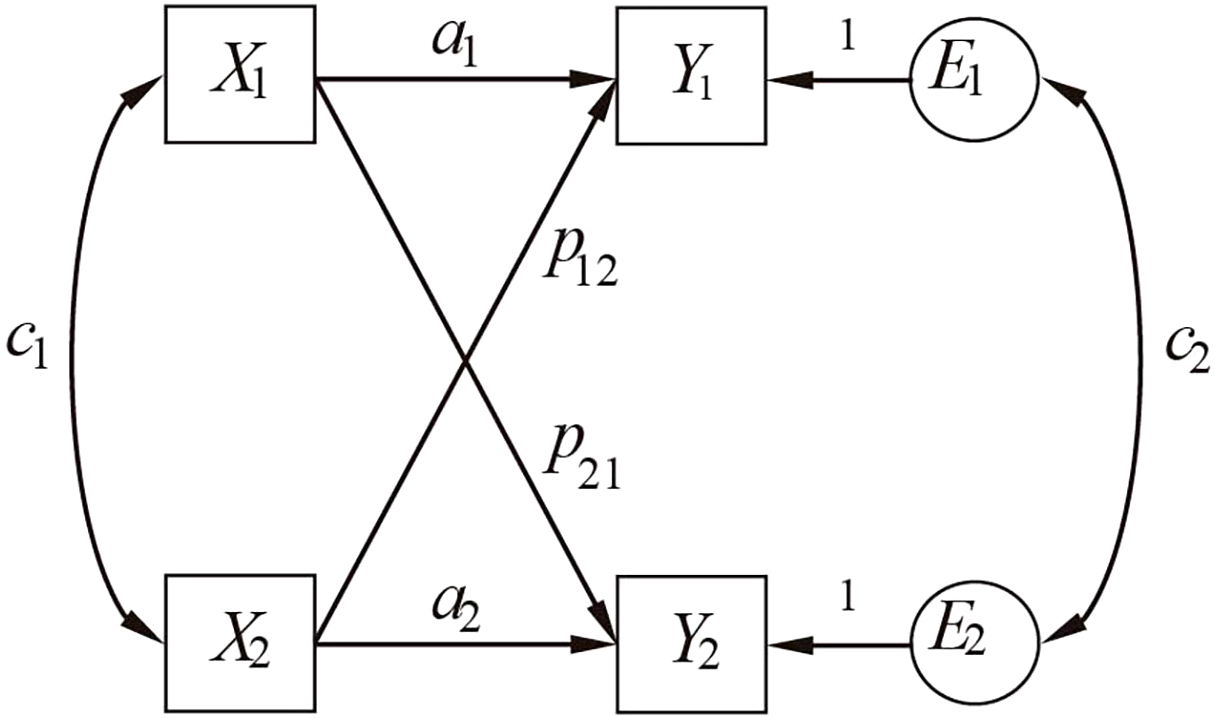
The APIM model with phantom variables. *P*_1_ /*P*_2_ = phantom variables for *k_1_*/*k_2_* estimation; *X*_1_ /*X*_2_, *Y*_1_ /*Y*_2_ = measured variables; *E*_1_ /*E*_2_ = error terms; *a*= actor effect; *p* = partner effect; *c*_1_ /*c*_2_ = correlated residuals.

Kenny et al. proposed an APIM method testing dyadic patterns using ratio *k* (partner effect/actor effect; *k* = *p*/*a*) (23). Indistinguishable dyads yield one *k* value (*p_12_*/*a_1_* or *p_21_*/*a_2_*), while distinguishable dyads yield two values of *k_1_* (*k_1_* = *p_12_*/*a_1_*) and *k_2_* (*k_2_* = *p_21_*/*a_2_*). To avoid division by zero and unstable estimates when actor effects are small, *k* should not be calculated if the standardized |*a*| < 0.10 (15). Critical *k* values are -1, 0, and 1 (24). Within the APIM framework, *k* and its confidence intervals (*CIs*) are directly estimable through phantom variables (*P*_1_ , *P*_2_)—latent constructs that preserve model fit (25). Mplus implementation (**Figure 2**) outputs *k_1_*/*k_2_* and *CIs* via predefined pathways.

#### 2.4.3 Pattern analysis steps

**Figure 3:**
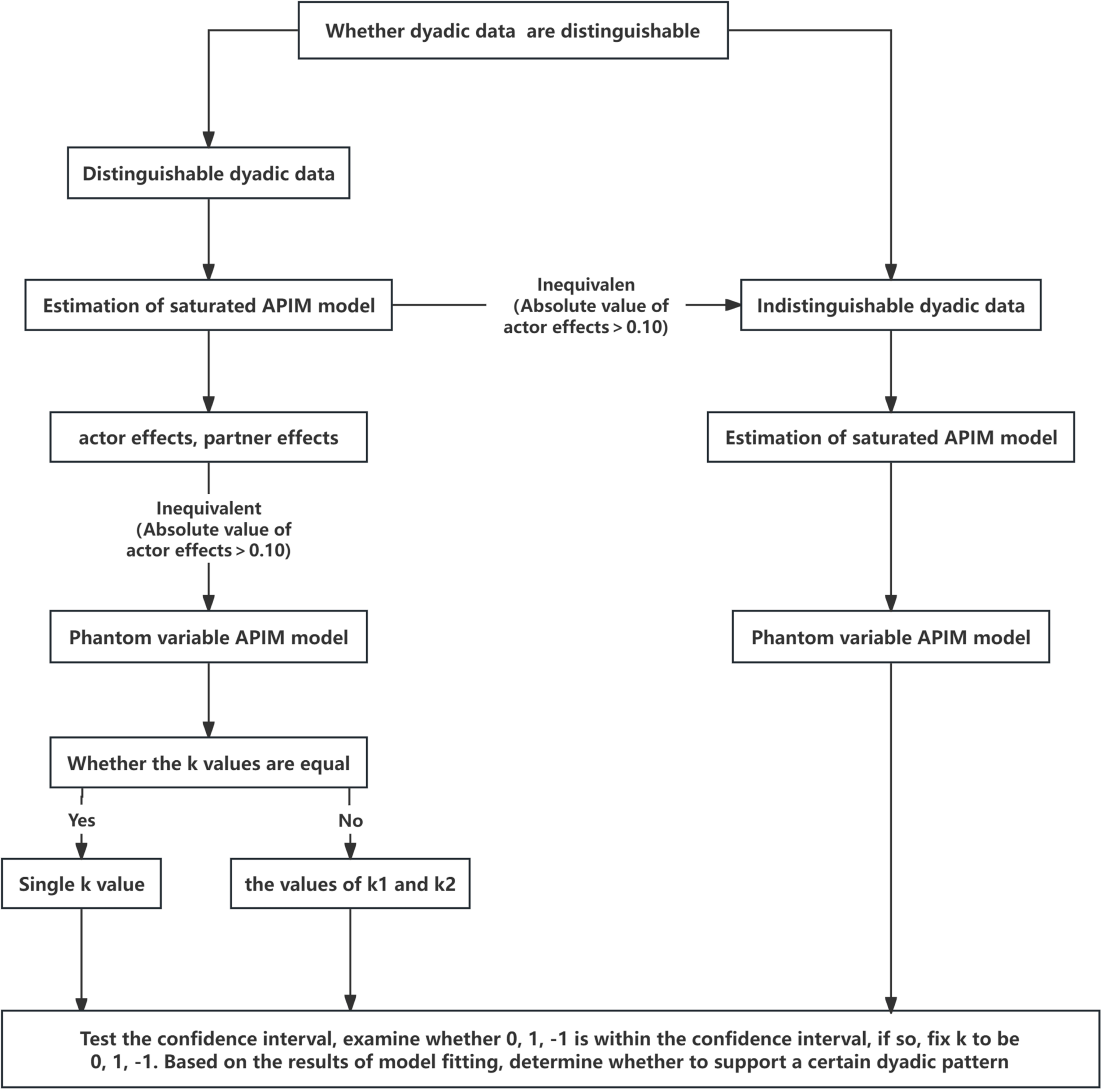
The flowchart illustrates the process of analysis of dyadic patterns within an APIM model using the Mplus. Excludes partner-only pattern due to low observed frequency.

Given partner-only configurations’ empirical rarity (26), analysis focused on actor-only, couple-oriented, and contrast patterns. Four sequential steps were implemented: first, estimating a saturated APIM to obtain actor (*a_1_*, *a_2_*) and partner effects (*p_21_*, *p_12_*); Second, testing actor effect equality (*a_1_* = *a_2_*) and partner effect equality (*p_21_* = *p_12_*). Dyadic data were treated as indistinguishable when *P* ≥ 0.2 (effects equal) or distinguishable when *P* < 0.2 (effects unequal); Third, calculating *k_1_* and *k_2_* separately via phantom variables. For distinguishable dyads, *k_1_* and *k_2_* equality was tested; non-significant differences warranted using a single *k*; Finally, Calculating *k*’s confidence interval (*CI*) and evaluating model fit to determine dyadic pattern: If *CI* included 0 but excluded ±1, *k* was constrained to 0 and the model re-estimated. Adequate fit indicated actor-/partner-only pattern; poor fit rejected both; If *CI* included 1 or -1, model fit determined support for couple-oriented or contrast patterns. For distinguishable dyads with equal *k* values, a common *k*’s *CI* determined the specific dyadic pattern (27) (**Figure 3**).

#### 2.4.4 Statistical analysis

Analyses were conducted using SPSS 26.0, Mplus 8.3 and R 4.5.0, with statistical significance set at *P* < 0.05. Continuous variables were expressed as medians (interquartile ranges) and categorical variables as frequencies (%). Differences in demographics, prenatal stress, and depression were assessed via wilcoxon signed ranks test and χ² tests. Internal consistency was evaluated using Cronbach’s α, while Pearson correlations examined prenatal stress-depression associations. Sensitivity analysis was performed using multiple imputation methods to assess the impact of missing data. Generalized linear models incorporating a stress-by-gender interaction term were employed to examine gender differences in the stress-depression relationship among pregnant women and their partners.

Structural equation modeling employed robust maximum likelihood estimation with 5,000 bootstrap iterations to calculate 95% *Cis*. Model fit was evaluated using multiple criteria: χ²/*df* ratio < 3, comparative fit index (CFI) ≥ 0.90, root mean square error of approximation (RMSEA) ≤ 0.08, standardized root mean square residual (SRMR) ≤ 0.08, and Tucker-Lewis index (TLI) ≥ 0.90 (28, 29).

## 3 Results

### 3.1 Demographic features of the participants

The sample comprised 282 couples with pregnant women averaging 31 years (mean gestation=39 weeks) and husbands averaging 33 years. Among pregnant women, those with undergraduate education constituted the highest proportion (37.9%). Pregnant women demonstrated higher prenatal stress scores and depression prevalence than husbands. Significant gender disparities emerged in both prenatal stress and depression levels (**Table 1**). Following data interpolation, a sensitivity analysis was conducted, which demonstrated robust results (**Supplementary Table 1**).

**Table 1:** Descriptive characteristics of the participants (N = 282).

Variables	Total (N=564)	Pregnant women (N=282)	Husbands (N=282)	*Z*/*χ^2^*	*P* value
**Age**	32 (29,35)	31 (28,34)	33 (30,36)	10.105	**< 0.001**^a^
**Prenatal stress**	24 (17,28)	24 (18,28)	22 (17,28)	-2.038	**0.042**^a^
**Gestation week**	-	39 (38,39)	-		
Education					
Below High School Level	-	41 (7.270)	-		
Undergraduate	-	214 (37.943)	-		
Graduate	-	17 (4.787)	-		
**Prenatal depression**	4 (1,2)	5 (2,9)	4 (1,6)	-3.477	**0.001**^a^
				7.058	**0.008**^b^
Depressed	89 (15.780)	56 (19.858)	33 (11.702)		
Not Depressed	475 (84.220)	226 (80.142)	249 (88.230)		
**Occupation**				5.064	**0.024** ^b^
with occupation	539 (95.567)	264 (93.617)	275 (97.518)		
without occupation	25 (4.433)	18 (6.383)	7 (2.482)		
**Family history of hypertension**				0.002	0.968^b^
Yes	104 (18.440)	52 (18.571)	52 (18.440)		
No	458 (81.206)	228 (81.429)	230 (81.560)		
**Family history of diabetes**				0.744	0.388^b^
Yes	50 (8.865)	28 (9.964)	22 (7.885)		
No	510 (90.426)	253 (90.036)	257 (92.115)		

a Comparisons between groups were made using wilcoxon signed ranks test and are presented as P_50_ (P_25_, P_75_);

b Comparisons between groups were made using the χ² test, and and are presented as frequencies (%). There are missing values for family history of hypertension and diabetes, with 2 and 4 cases missing.

The bold values indicate statistical significance (*P*<0.05).

### 3.2 Correlation analysis of variables

The results indicated significant positive correlations between prenatal stress and prenatal depression in both individuals and their partners. Specifically, pregnant women’s and, husbands’ stress levels correlated with their own depression and their partners’ depression (**Table 2**).

**Table 2:** Correlational analysis of prenatal stress and prenatal depression in couples (N = 282).

Variables	Prenatal stress in pregnant women	Prenatal stress in husbands	Prenatal depression in pregnant women	Prenatal depression in husbands
Prenatal stress in pregnant women	1			
Prenatal stress in husbands	0.212^**^	1		
Prenatal depression in pregnant women	0.629^**^	0.168^**^	1	
Prenatal depression in husbands	0.132*	0.482^**^	0.208^**^	1

*P< 0.05, **P < 0.01.

### 3.3 APIM analyses

#### 3.3.1 The fitness index of APIM model

Analysis commenced with a saturated APIM. Constraining two actor and two partner effects revealed significant between-couple path discrepancies (*P* < 0.20), establishing dyadic distinguishability. Phantom variable integration yielded *k_1_* and *k_2_* estimates with 95% CIs encompassing zero. Parameter fixation (*k_1_* = *k_2_* = 0) produced a well-fitting model demonstrating excellent conformity: χ²/*df*=1.423, SRMR=0.016, CFI=1.000, TLI=1.012, RMSEA=0.000, all meeting established thresholds (**Table 3**).

**Table 3:** The APIM model fit index.

Model	*df*	*χ^2^*	*χ^2^*/*df*	SRMR	RMSEA	TLI	CFI
Saturated APIM model	0.000	0.000	0.000	0.000	0.000	1.000	1.000
Phantom variables APIM model	0.000	0.000	0.000	0.000	0.000	1.000	1.000
APIM model with a fixed k value of 0	2,000	0.949	0.475	0.016	0.000	1.012	1.000

*df*, degree of freedom; *χ^2^*, chi-square; SRMR, standardized root mean square residual; CFI, comparative fit index; TLI, tucker lewis index; RMSEA, root mean square error of approximation.

#### 3.3.2 The APIM analysis of prenatal stress and prenatal depression in couples

**Figure 4:**
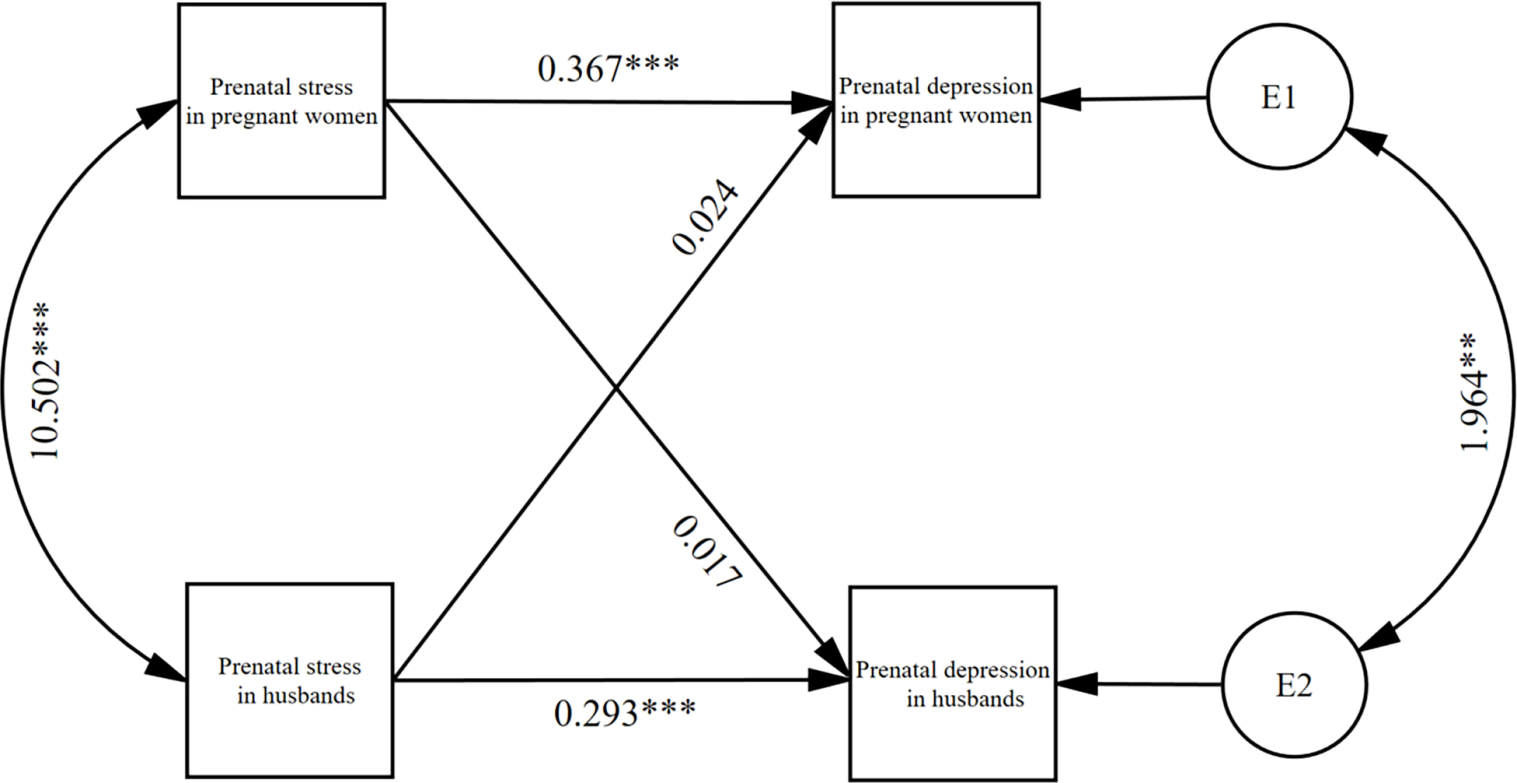
the results of the APIM of prenatal stress and prenatal depression in couples (N=282). *P< 0.05, **P < 0.01, ***P < 0.001.

**Figure 4** presents the constrained model with effect values detailed in **Table 4**. The analysis revealed *k_1_* = 0.064, 95% *CI:* (-0.113, 0.260) and *k_2_* = 0.064, 95% *CI*: (-0.118, 0.249). The confidence intervals for both *k_1_* and *k_2_* included zero, indicating an actor-only pattern in the APIM. Specifically, prenatal stress positively predicted one’s own prenatal depression but did not significantly influence the partner’s depression.

**Table 4:** The results of effect estimation and k values in APIM during couples.

	Actor effect	*P* value	Partner effect	*P* value	*k* value	95% *CI*
Pregnant women	0.367	<0.001	0.017	0.482	0.064	[-0.113, 0.260]
Husbands	0.293	<0.001	0.024	0.520	0.059	[-0.118, 0.249]

*k*=*p*/*a*, where *a* represents the actor effect and *p* represents the partner effect.

At the same time individual prenatal stress had a statistically significant positive effect on prenatal depression (*β* = 0.367, *P* < 0.001; *β* = 0.293, *P* < 0.001), and no effect on partner prenatal depression (*β* = 0.024, *P* = 0.482; *β* = 0.017, *P* = 0.520). Specifically, an elevated risk of prenatal depression was observed in both pregnant women and their husbands as prenatal stress levels increased.

To investigate gender differences in the stress-depression relationship among pregnant women and their partners, a generalized linear model incorporating a stress-by-gender interaction term was employed. The interaction term did not attain statistical significance (*β* = 0.074, *P* = 0.081), indicating no evidence for gender moderation of the stress-depression relationship within partner dyads (**Supplementary Table 2**).

## 4 Discussion

This study demonstrated elevated prenatal depression prevalence, with 20% among pregnant women exceeding international reports (7.4-12.8%) (30) yet aligning with domestic studies (3.6-40.2%) (31–33). And 12% among expectant fathers, higher than meta-analytic estimates (9.8%) (8). These cross-study discrepancies likely reflect methodological heterogeneity in screening tools, and population characteristics. Notably, this study revealed significant gender disparities in prenatal stress and depression among expectant parents. Pregnant women demonstrated markedly higher prenatal stress scores and a greater prevalence of prenatal depression compared to their husbands, aligning with previous research findings (34). The observed gender gradient may stem from synergistic mechanisms: physiological adaptations (gestational changes) (35), psychosocial stressors (role transitions) (36), and neurocognitive factors (enhanced emotional recall influencing symptom reporting) (37), collectively amplifying stress-depression associations (8). These findings underscore the necessity for gender-specific mental health interventions during perinatal care.

The present cross-sectional study investigated the APIM of prenatal stress and depression within couples, revealing an actor-only pattern. Specifically, individual prenatal stress influences only one’s own prenatal depression, without affecting the spouse’s. Notably, higher maternal and spousal stress as a risk factor for individual prenatal depression, aligning with previous research (38). Stress may lead to depression via mechanisms such as hypothalamic-pituitary-adrenal (HPA) axis dysregulation (39), neurotransmitter imbalances (40), neurotrophic factor inhibition (41). Consequently, maintaining a positive attitude and managing stress effectively are crucial for both pregnant women and their partners to reduce the risk of prenatal depression.

Family systems theory posits that individuals’ mental health is influenced by family interactions and emotional bonds (16). Notably the study did not detect a significant impact of individual prenatal stress on spouses’ prenatal depression, a finding that might stem from traditional Chinese gender - role expectations. In traditional Chinese culture, men are typically seen as the breadwinners, while women are expected to manage domestic matters. These roles can shape different perceptions, emotional reactions, and coping mechanisms when couples encounter stress (42), potentially explaining the lack of partner pattern. Adjusting gender - role attitudes may enhance couples’ ability to handle prenatal stress (43). Moreover, collectivism and family harmony are central to Chinese culture, deeply influencing couples’ interactions (44). To preserve harmony, couples might suppress negative emotions and deal with stress independently instead of turning to their partners, leading to less emotional sharing and weakened mutual support (45). Finally, pervasive mental health stigma (46) may further reinforces this tendency. Future research could explore these cultural dynamics more deeply to better understand their impact on couple interactions and stress management.

Currently, there is a scarcity of studies, both domestically and internationally, that explore the correlation between prenatal stress and prenatal depression within the context of couples. Utilizing dichotomous dyadic couple data and the latest APIM, this study revealed an actor-only pattern for prenatal stress and depression within relationships. Consequently, we recommend that healthcare providers concurrently implement standardized psychological screening (e.g., PSS, PHQ-9, EPDS) for both expectant mothers and fathers during routine prenatal visits to enable early detection of significant stress or depression risk in either partner. Practical tips for couples to cope with stress together should also be integrated into prenatal education programmes and partner counselling to improve the ability of couples to communicate about their feelings of stress and solve problems together. In addition, individualised psychological support should be provided to either partner with emotional distress, such as cognitive behavioural therapy to help them adjust their negative thinking. These strategies mitigate parental psychological distress, reduce prenatal depression risk, and ultimately enhance pregnancy and postnatal outcomes.

However, several limitations should be acknowledged in this study. Although anonymous surveys, confidentiality protocols, and reliable self-administered scales were implemented, recall and social desirability biases remain unavoidable. Future research should incorporate objective measures (e.g., cortisol levels) to validate self-reports. The cross-sectional nature of the study precluded causal inferences and limited the assessment to the psychological state of couples in late pregnancy. Longitudinal study designs with regular data collection are recommended for future research to better capture the temporal evolution and interplay of stress and depression. Notably, the sample was drawn from a single hospital in Fuzhou City. While this hospital, undertaking most of Fuzhou’s maternal deliveries, partially reflects the mental health of local pregnant women, the findings may not generalize to pregnant populations in other geographic, socioeconomic, or healthcare settings. Future studies should diversify recruitment regions to enhance the generalizability of results.

## 5 Conclusion

Prenatal stress and prenatal depression in couple dyads fall within an actor-only pattern of the APIM, individual prenatal stress affects only one’s own prenatal depression and not that of one’s spouse. The higher the stress of the pregnant woman and her spouse, the higher the risk of individual prenatal depression. Healthcare professionals and family members should pay attention to the prenatal stress of pregnant women and their husbands at the same time, and reduce the couple’s prenatal stress through comprehensive interventions, so as to prevent and alleviate the occurrence of prenatal depression in both partners, and to improve pregnancy outcomes and postnatal health.

## Data Availability

The raw data supporting the conclusions of this article will be made available by the authors, without undue reservation.

## Supplementary material

Supplementary Table 1 Descriptive characteristics of the participants.

Supplementary Table 2 Gender differences in the stress-depression relationship in couples.
