# Correction: The interplay of prenatal stress and prenatal depression in Chinese couples: based on the actor-partner interdependence model

**DOI:** 10.3389/fpsyt.2025.1682329

**Published:** 2025-08-20

**Authors:** Frontiers Production Office

**Affiliations:** Frontiers Media SA, Lausanne, Switzerland

**Keywords:** actor-partner interdependence model (APIM), patterns, prenatal stress, prenatal depression, couples’ mental health

Author Jungu Zhou was erroneously omitted as equal contributing author.

There was an error in the keywords. “Couples’ mental” should be “Couples’ mental health”.

The ethical approval number was erroneously given as 2023KY117. The correct approval number is 2020KY117.

The original version of this article has been updated.

