# Supplementary material for: The interplay of prenatal stress and prenatal depression in Chinese couples: based on the actor-partner interdependence model
Source: Front Psychiatry. 2025 Jul 31;16:1607470. doi: 10.3389/fpsyt.2025.1607470 (PMC12352324; doi:10.3389/fpsyt.2025.1607470)
Supplement: Supplementary Table 1 — Descriptive characteristics of the participants. [file Table1.docx]

**Supplementary Material**

**1 Supplementary Tables**

Supplementary Table 1 Descriptive characteristics of the participants.

| **Variables** | **Total(N=564)** | **Pregnant women(N=282)** | **Husbands(N=282)** | ***Z*/*χ^2^*** | ***P* value** |
| --- | --- | --- | --- | --- | --- |
| Age | 32(29,35) | 31(28,34) | 33(30,36) | 10.105 | **< 0.001^a^** |
| Prenatal stress | 24(17,28) | 24(18,28) | 22(17,28) | -2.038 | **0.042^a^** |
| Gestation week | - | 39(38,39) | - |  |  |
| Education |  |  |  |  |  |
| Below High School Level | - | 41(7.270) | - |  |  |
| Undergraduate | - | 214(37.943) | - |  |  |
| Graduate | - | 17(4.787) | - |  |  |
| Prenatal depression | 4(1,2) | 5(2,9) | 4(1,6) | -3.477 | **0.001^a^** |
|  |  | | | 7.058 | **0.008^b^** |
| Depressed | 89(15.780) | 56(19.858) | 33(11.702) |  |  |
| Not Depressed | 475(84.220) | 226(80.142) | 249(88.230) |  |  |
| Occupation |  | | | 5.064 | **0.024^b^** |
| with occupation | 539(95.567) | 264(93.617) | 275(97.518) |  |  |
| without occupation | 25(4.433) | 18(6.383) | 7(2.482) |  |  |
| Family history of hypertension |  |  |  | 0.012 | 0.914^b^ |
| Yes | 105(18.617) | 53(18.794) | 52(18.440) |  |  |
| No | 459(81.383) | 229(81.206) | 230(81.560) |  |  |
| Family history of diabetes |  |  |  | 0.539 | 0.463^b^ |
| Yes | 51(9.043) | 28(9.929) | 23(8.156) |  |  |
| No | 513(90.957) | 254(90.071) | 259(91.844) |  |  |

*Note.* ^a^ Comparisons between groups were analyzed using the Wilcoxon signed-rank test and are presented as P_50_ (P_25_, P_75_), ^b^ comparisons between groups were made using the χ² test, and are presented as frequencies (%). The bolded effect sizes indicate statistical significance (*P*<0.05).

**Supplementary Table 2 Gender differences in the stress-depression relationship in couples**

| **Variables** | ***β*** | ***P* value** | ***OR* (*95% CI*)** |
| --- | --- | --- | --- |
| Stress | 0.297 | **< 0.001** | (0.235,0.359) |
| Gender | 0.776 | **0.011** | (0.181,1.371) |
| Stress*Gender | 0.0740 | 0.081 | (-0.009,0.159) |
